# Exploring the Potential of Phytogenic Materials for Bone Regeneration: A Narrative Review of Current Advances and Future Directions

**DOI:** 10.7759/cureus.48175

**Published:** 2023-11-02

**Authors:** Turki M Abu Alfar, Wedad S Alaida, Hassan A Hammudah, Lamis L Mohamado, Riyam R Gaw, Lamia Al-Salamah, Bayan A Alasmari, Rawan M Alotaibi, Mona A Almutairi

**Affiliations:** 1 Periodontics, National Guard Hospital, Madinah, SAU; 2 Prosthodontics, Ministry of Health, Riyadh, SAU; 3 Dentistry, Taibah University, Madinah, SAU; 4 Dentistry, Ministry of Health, Dammam, SAU; 5 Periodontics, Ministry of Health, Riyadh, SAU; 6 Research and Studies, Ministry of Health, Riyadh, SAU; 7 Endodontics, Ministry of Health, Riyadh, SAU

**Keywords:** periodontal flap surgery, novel bone graft substitute, phytogenic materials, bone graft substitutes, periodontal surgery

## Abstract

In dentistry, bone regeneration in areas following tooth loss, the removal of a tumor or cyst, and craniofacial surgery can be accomplished by using bone grafts. Many biocompatible materials have been employed for bone regeneration in dentistry; however, all these bone graft materials come with various drawbacks. Therefore, there is a growing demand for natural, cost-effective, and biocompatible plant-based bone grafts. This review explores the emerging field of phytogenic elements in bone restoration and their specific applications in dentistry. The review focuses on key phytogenic compounds, such as algae-based and plant-based bone substitutes, delineating their roles in bone regeneration in dental bone defects. It also highlights the existing challenges associated with phytogenic grafts, such as limited bioavailability and high-dose toxicity. This calls for increased research into compatible, affordable carriers and a broader spectrum of studies to determine the most effective phytogenic solutions in dental regenerative medicine.

## Introduction and background

A bone typically undergoes natural regeneration on its own, unless factors such as necrosis, degenerative bone disorders, tumors, cystic lesions, and malunion are involved. In such cases, the need for surgery and the placement of bone grafts are common approaches to restoring bone defects. Various specialties, such as orthopedics, dentistry, and oncology, use bone grafts for surgical bone regeneration. Over 2 million bone grafting procedures are performed annually, making the bone the second most transplanted tissue after blood transfusions [1]. In dentistry, bone regeneration in regions following tooth loss, removal of a tumor or cyst, and craniofacial surgery can be achieved by using bone grafts. Similarly, bone grafts are a prerequisite for successful implant therapy in areas of inadequate bone availability. A bone graft is medically described as living tissue with the inherent capacity to stimulate bone healing. It is strategically introduced into a bony defect, either independently or in conjunction with supplementary materials [2]. Bone grafts are used to provide physical support and stimulate osteogenesis, leading to bone replacement. There are four fundamental biological properties that are vital for effective bone regeneration. These include osseointegration, osteogenesis, osteoconduction, and osteoinduction [3]. Osseointegration is defined as the process by which a grafting material chemically bonds with the surface of the bone, establishing a direct connection devoid of fibrous tissue. Osteogenesis entails the formation of new bone tissue through the activity of osteoblasts, or progenitor cells, present within the grafting material. Osteoconduction, on the other hand, refers to the property of a bone grafting material to serve as a scaffold, facilitating the growth of host cells [4]. Osteoinduction is characterized by the recruitment of host-derived stem cells to the grafting site, where local proteins and various bioactive factors play a pivotal role in promoting the differentiation of these recruited stem cells into osteoblasts [5]. Growth factors like platelet-derived growth factors (PDGFs), fibroblast growth factors (FGFs), and transforming growth factors (TGFs) influence the process of bone formation [6,7].

Currently, existing bone grafts and substitute materials primarily function as a framework to support bone regeneration processes, thereby fulfilling the osteoconductive aspect [8]. Other natural grafts used in dentistry are autografts, allografts, xenografts, and phytogenic materials. Autologous or autogenous bone grafting involves the use of bone obtained from the same individual who received the graft. Allografts are harvested from individuals other than those who received the grafts. It was obtained from cadavers who had donated their bones. Xenografts are bone grafts from species other than humans, such as bovines, and are used as calcified matrices [9]. Although there are different bone grafts available for promoting bone formation, their application in clinical settings is limited due to their cost, the need for technique-sensitive instrumentation, associated risk, and inconsistent results [10]. Phytogenic materials, such as gusuibu and grafts based on marine algae, represent bone graft materials sourced from plants [2]. Phytogenic materials are easily available, cost-effective, possess less risk of rejection by the human body or transmission of any infection, and most importantly, are abundantly available in nature. Therefore, these plant-based biocompatible materials can be considered as bone grafts in the future, increasing their scope for further investigations and studies.

The aim of this study is to survey the literature concerning various plant-based bone grafts, examining their mechanisms of action, sustainability, instrumentation methodologies, and associated success rates in bone regeneration. Presently, the available literature in English on this topic is sparse, posing a challenge to garner a comprehensive understanding.

## Review

Research methodology

We carried out an extensive search across recognized databases, including PubMed, Google Scholar, Excerpta Medica database (Embase), and Medical Literature Analysis and Retrieval System Online (MEDLINE), using keywords relevant to plant-based bone grafts and their applications. The extraction process prioritized pertinent clinical trials, systematic reviews, and meta-analyses. Our inclusion criteria were limited to studies published exclusively in English. In the preliminary phase of our selection, we excluded articles based on a cursory review of their abstracts. This was followed by an in-depth review of the remaining abstracts, further refining our selection to only those studies that directly addressed our research objectives.

Criteria for an ideal bone graft

An ideal bone graft, as highlighted by Wang W et al. [3], should provide mechanical support and stimulate bone regeneration, encompassing four fundamental biological properties: osseointegration, osteogenesis, osteoconduction, and osteoinduction. Such a graft should be easily harvested and fit the desired site in the appropriate shape and quantity. Additionally, it should carry a minimal risk for disease transmission and be cost-effective [11-12].

Phytogenic materials

Phytogenic materials, a subcategory of natural bone graft substitutes that also include allografts, autografts, and xenografts, originate from plants and are used as bone replacement materials. Gusuibu and marine algae stand out as known sources, but a plethora of other plant-derived alternatives exist. In vitro research has highlighted that these phytogenic materials can stimulate stem cell differentiation toward osteogenesis and exhibit angiogenic potential. Tissue engineering techniques permit the incorporation of plant-derived compounds or extracts into biomaterials. However, mainstream acceptance of phytogenic materials grapples with obstacles such as a lack of predictive utilization, doubts about clinical efficacy, and concerns related to quality control standards [2].

Types of phytogenic materials

Phytogenic materials can be broadly divided into two types: algae-based substitutes (e.g., Algipore^TM^) and plant-based substitutes (e.g., gusuibu, icariin, naringin, quercetin, kaempferol, puerarin, curcumin, berberine, resveratrol, salvianolic acids, ginsenosides, and ursolic acid).

Algae-Based Bone Graft Substitutes

Certain marine algae consisting of naturally occurring hydroxyapatite (HA), Algipore^TM^, have been used clinically as bone substitute materials since 1988. This plant-based material has the ideal properties of a bone graft, such as low immunogenicity, better adhesion to proteins, slow resorption, and is biocompatible. They also act as carriers of growth factors (GFs) and mesenchymal stem cells (MSCs). A 14-year retrospective study conducted by Ewers et al. evaluated 209 sinus grafts performed on 118 patients with a severely resorbed maxillary alveolar process. The results showed a 95% implant success rate following sinus grafting using Algipore^TM^ [13].

Plant-Based Bone Graft Substitutes

Traditional Chinese medicine (TCM), derived from herbs and plant extracts, has long been utilized by the Chinese population, demonstrating potential for bone regeneration. These accessible and cost-effective resources represent a key area of research in plant-based bone graft substitutes [14]. However, the majority of such medicines are administered orally, distributing the drug throughout the body rather than targeting a specific area, raising concerns of systemic toxicity, kidney and liver complications, and other adverse reactions. Shi G. et al. suggest that combining TCMs with different biomaterials can enhance targeted drug delivery and bone regeneration, reducing toxicity risks [15]. They emphasize that current research primarily relies on small-scale preclinical or animal studies, advocating for more extensive studies and human clinical trials. Notably, there's a marked gap in research on plant graft substitutes beyond the TCM realm.

*Gusuibu *(*Rhizoma Drynariae*)

In TCM, gusuibu is widely used for the treatment of bone fractures and osteoarthritis [2]. Gusuibu is the dried rhizome of the perennial pteridophyte *Drynaria fortunei*. This material has osteoinductive properties and increases alkaline phosphatase activity, which promotes bone remodeling and calcification. In 2006, Wong and Rabie conducted a study on 14 New Zealand white rabbits. They created 20 defects in the rabbit skulls. They found that when Gusuibu was combined with a collagen carrier, serving as a structural scaffold, new bone formation across the bony defect increased by 24% compared to using grafted Gusuibu alone. Furthermore, it increased by 90% when compared to an absorbable collagen sponge that is typically used as a carrier for growth factors (GFs) such as bone morphogenetic proteins (BMPs) [16]. Gusuibu has been shown to accelerate bone remodeling following orthodontic tooth movement by promoting osteoblastic activity, and cell culture studies further reveal that it can regulate both osteoclast and osteoblast activities, aiding in bone remodeling [14,17].

*Icariin *(*Epimedium pubescens*)

Icariin, a flavonoid compound, is derived from the Chinese botanical source known as *Epimedium pubescens*. This flavonoid has enhanced osteoblast proliferative properties and can inhibit the transformation of osteoclasts into adipocytes, thereby promoting bone formation and reducing bone resorption [15]. Gürbüz et al., in 2019, conducted a study on rats, where icariin, when administered locally to the fracture site, showed accelerated bone healing by reducing oxidative stress [18].

However, icariin has major drawbacks of very low bioavailability and a short half-life of one to two hours. As bone regeneration takes approximately three to six months, the use of icariin has become challenging due to its short half-life. Therefore, researchers have considered adding an appropriate carrier so that there is long-term and stable release of the drug at the affected site. Considering this, icariin was combined with a calcium phosphate-based biomaterial, and both in vivo and in vitro studies showed that icariin combined with calcium phosphate contributed to bone regeneration and angiogenesis, as well as overcoming the challenges associated with the use of icariin alone by promoting bioavailability and long-term stable drug release. Icariin has also been combined with other materials, such as bioglass or gelatin, nanofibrous membranes, and chitosan/hydroxyapatite scaffolds, all of which showed improved osteointegration [15].

Naringin

Naringin constitutes the principal constituent of *Drynaria fortunei*, a type of fern. Furthermore, it is present in various citrus fruits, vegetables, tomatoes, and grapefruits. In a study conducted by Wu et al. in 2008, naringin, a flavonoid, was used in osteoporosis and showed enhanced alkaline phosphatase activity, osteocalcin levels, osteopontin synthesis, and cell proliferation in primary cultured osteoblasts, thereby proving that it helps in bone regeneration. [19] The limitation of naringin is like that of icariin; when taken orally, it exhibits low bioavailability because of its poor water solubility and dissolution rates. Therefore, naringin must be combined with biomaterials to increase its bioavailability and prevent its degradation. In 2006, Wong and Rabie grafted naringin with a collagen matrix carrier into full-thickness parietal bone defects in rabbits. Histological analysis at two weeks showed that Naringin demonstrated superior efficacy in promoting early bone remodeling and bone formation compared to autogenous endochondral bone grafts alone and collagen matrices alone. Similarly, a study by Chen K et al. (2013) found that rabbit calvarial defect models implanted with naringin-loaded porous biodegradable gelatin/β-tricalcium phosphate (β-TCP) composites showed enhanced ingrowth of new bone into the defect site. Histological examination from this study revealed complete osseointegration of the biodegradable implant, with newly generated bone replacing a substantial portion of the composite material by 8 weeks [20-21].

Quercetin

Quercetin is a flavonoid commonly found in compounds of Chinese medicines, such as those derived from *Sambucus williamsii*, and is also present in various vegetables and fruits. It promotes osteoblastic cell proliferation, differentiation, and mineralization, inhibits osteoclastic cell proliferation and maturation, and further enhances antioxidant expression while curbing oxidative stress [15]. However, its drawbacks align with those of icariin and naringin, which necessitates its combination with other materials. To bolster its sustainability and efficacy, quercetin has been incorporated into collagen matrices, HA scaffolds, and calcium-deficient hydroxyapatite (CDHA), demonstrating a sustained release spanning 14 to 120 days, depending on the materials utilized. A study by Raja N et al. in 2021 concluded that, over a test period of 120 days, quercetin-loaded composites could achieve a sustained release of quercetin without any initial burst [22].

Kaempferol and Puerarin

Kaempferol is a flavonoid found in *Kaempferia galangal L.*, *Ginkgo biloba L.*, *Thesium chinense T.*, aloe vera, *Rosmarinus officinalis*, *Hippophae rhamnoides L.*, and hawthorn, whereas puerarin is derived from the roots of *Pueraria lobata* (gegen). In accordance with findings from a multitude of in vitro and animal investigations, both kaempferol and puerarin have shown positive osteogenic properties that enhance bone health. Similar to the other flavonoids discussed earlier, these two also have to be combined with other materials for better action and bioavailability [15].

Curcumin

Curcumin is an alkaloid compound found predominantly in *Curcuma longa Linn* and in plants of the ginger family. Turmeric is the most common source of curcumin and is abundantly available in nature. Curcumin has antioxidant and anticancer properties; in addition to that, it can also increase the proliferation of osteoblasts, affect osteoclastic activity, and inhibit bone resorption [15]. A study revealed that curcumin when taken orally, can successfully close extensive defects and facilitate the restoration of bone tissue surrounding titanium implants in diabetic rats induced with streptozotocin [23].

Curcumin has reduced bioavailability owing to its low aqueous solubility, extremely rapid systemic elimination, and insufficient tissue absorption and degradation, but has been shown to provide adequate bioavailability and sustained release of curcumin into the system [15]. In 2020, Ghavimi M.A. et al. developed a guided bone regeneration membrane infused with curcumin and aspirin. When this membrane was placed in the jaw defects of dogs, it demonstrated enhanced proliferation and differentiation of dental pulp stem cells (DPSC). Furthermore, there was elevated alkaline phosphatase (ALP) activity and increased expression of osteoblastic genes, such as RUNX2 and osteocalcin (OCN). Complete bone regeneration was observed within 28 days of implantation, as confirmed through histological examination. In contrast, the area with the commercial membrane remained unoccupied [24].

Resveratrol, Salvianolic Acids, Ginsenosides and Ursolic Acid

Resveratrol, salvinolic acids, ginsenosides, and ursolic acid all exhibit osteogenic properties. They promote osteoblast proliferation and differentiation and concurrently inhibit osteoclast differentiation through various signaling pathways. Notably, in in vitro studies, these compounds are primarily not used alone; they are often compounded with other materials. Despite the promising results shown in animal models, human trials are essential to confirm their true efficacy in bone regeneration [15].

Morinda Citrifolia (Noni) Fruit Extract 

*Morinda citrifolia*, commonly known as noni or Indian mulberry, is a tropical fruit of the Rubiaceae family. Historically, it has been used for diverse medicinal purposes, including strengthening bones and boosting immunity, as well as treating conditions like osteoporosis, depression, and inflammatory diseases such as rheumatoid arthritis and dermatitis [25-26]. Recent studies have highlighted the fruit extract's beneficial properties, marking it as a potential bone regenerative material [26-27]. Reinforcing this, research has shown enhanced wound-healing activity in animals treated with *Morinda citrifolia* extract [28]. Building on its potential in bone regeneration, a randomized clinical trial by Sabu et al. examined the use of *Morinda citrifolia* fruit extract in open-flap debridement surgery. The fruit was processed into powder, applied to the defect, and evaluated using cone-beam computed tomography systems (CBCT). The outcome indicated a remarkable 27.67% improvement in the group treated with the noni extract, against a 14.50% improvement in the control group [29].

Other Plant-Based Materials in Combination With Biomaterials

In recent findings, spherical gold nanomaterials with a diameter of 71.5 nanometers, synthesized using the aqueous bark extract of Salacia chinensis, have emerged as promising candidates for dental implant applications. A study conducted by Jadhav et al. in 2018 demonstrated that these photosynthesized nanomaterials exhibit cytocompatibility and compatibility with blood components, including periodontal fibroblasts and erythrocytes. Additionally, the gold nano biomaterial was found to enhance the viability of human MG-63 bone osteosarcoma cell lines, suggesting its potential for promoting osteoinduction. This property holds promise for its application in dental graft treatments as a “bone inductive agent. [30]

In a 2019 study by Lee J. et al. [31], osteoblast-seeded apple scaffolds were grafted into rat calvarial defect models to assess bone regeneration. After implantation, rats were sacrificed at intervals of two, four, and eight weeks for serial evaluations. Micro-computed tomography (CT) scans and visual inspections at eight weeks post-grafting revealed partial regenerative growth in the implanted regions. Cell formation and angiogenesis were observed within the scaffold areas. Moreover, human induced pluripotent stem cells (iPSCs)-derived osteoblasts (hiPSC-Obs) were detected on the scaffolds, indicating their survival on a vegetal scaffold in an in vivo environment. The research emphasizes the potential of specific scaffold types, like porous apples, as bone grafts [31-32].

*Cissus quadrangularis*, recognized for its bone regeneration potential, was systemically administered in a study by Altaweel et al. The results underscored its ability to speed up mandibular bone formation and boost bone density, suggesting its potential application as a graft material or in combination with other graft materials [33]. All studies and investigations have been consolidated in Table 1.

**Table 1:** Various studies conducted in the literature on phytogenic bone grafts

S.No.	Author & year	Phytogenic bone graft used	Study	Conclusion
1.	Ewers et al., 2005 [13]	Algipore^TM^	209 sinus grafts using Algipore^TM^ were performed on 118 patients who presented with a severely resorbed maxillary alveolar process.	A 95% implant success rate following sinus grafting procedures with AlgiPore^TM^ was found.
2.	Wong and Rabie, 2006 [16]	Naringin	Naringin with a collagen matrix carrier was grafted into full-thickness parietal bone defects in rabbits and was analyzed after two weeks.	Naringin demonstrated superior early bone remodeling and bone formation in comparison to both autogenous endochondral bone grafts and collagen matrices.
3.	Gürbüz K et al., 2019 [18]	Icariin	Icariin was applied to a femoral fracture in 64 male rats and was evaluated with radiography, histopathology, and dual-energy X-ray absorptiometry. Activities of superoxide dismutase (SOD), glutathione peroxidase (GPx), glutathione (GSH), and myeloperoxidase (MPO) levels were measured in the peripheral blood.	Icariin, when administered locally to the fracture site, showed accelerated bone healing. Superoxide dismutase activity decreased in association with local icariin application to the fractured side, whereas GPx and GSH increased and MPO remained unchanged, thus reducing oxidative stress.
4.	Wong and Rabie, 2006 [20]	Gusuibu	20 skull defects were created on 14 New Zealand white rabbits. These defects were filled with gusuibu, gusuibu-integrated collagen, and collagen alone.	New bone formation was increased by 24% when a gusuibu-integrated collagen scaffold was used compared with the grafted gusuibu alone and by 90% when compared with an absorbable collagen sponge.
5.	Chen et al., 2013 [21]	Naringin	Rabbit calvarial defect models were implanted with a naringin-loaded porous biodegradable gelatin/β-tricalcium phosphate (β-TCP) composite.	The radiographic assessment revealed increased new bone ingrowth at the defect site, while histological examination demonstrated significant replacement of the composite material with newly formed bone at eight weeks.
6.	Raja N et al., 2021 [22]	Quercetin	A unique coiled-structured bioceramic calcium-deficient hydroxyl apatite (CDHA) containing quercetin was fabricated and cultured in cell culture media.	The quercetin-loaded composite could achieve sustained release of quercetin without any initial burst for a test period of 120 days.
7.	Cirano et al., 2018 [23]	Curcumin	One hundred rats were divided into five groups: diabetes mellitus (DM) + placebo (PLAC), DM + curcumin (CURC), DM + insulin (INS), DM + CURC + INS, and non-DM. In all groups, calvarian defects were created, and titanium implants were inserted into the tibia.	The DM + CURC + INS and non-DM groups exhibited greater closure of the calvaria compared to the DM + PLAC group, and increased retention of implants was observed in the DM + CURC, DM + CURC + INS, and non-DM groups when compared to the DM + PLAC group.
8.	Ghavimi M. A et al., 2020 [24]	Curcumin	A guided bone regeneration membrane loaded with curcumin and aspirin was developed and this membrane was placed in the jaw defect of dogs.	Accelerated proliferation and differentiation of dental pulp stem cells (DPSCs), along with elevated alkaline phosphatase (ALP) activity and increased expression of osteoblastic genes and associated proteins, led to full bone regeneration within only 28 days of implantation.
9.	Sabu et al. 2021 [29]	*Morinda citrifolia* Fruit Extract (noni)	Experimental group: patients with periodontal defects treated with powdered *Morinda citrifolia* fruit.	Cone-beam computed tomography systems (CBCT) assessments revealed a 27.67% bone volume improvement in the experimental group, compared to 14.50% in the control group.
10.	Jadhav K et al., 2018 [30]	Salacia chinensis	Aqueous bark extract of *Salacia chinensis* was combined with gold to create gold nanoparticles (GNPs), and their biocompatibility was studied.	The study demonstrated that phytosynthesized nanomaterials exhibit cytocompatibility and compatibility with periodontal fibroblasts and erythrocytes, suggesting that GNPs possess a heightened osteoinductive potential.
11.	Lee J et al., 2019 [31]	Osteoblast-grafted apple	Osteoblast-seeded apple scaffolds were grafted into rat calvarial defect models. The rats were sacrificed at two, four, and eight weeks for evaluation.	Micro-computed tomography (CT) and visual inspection at eight weeks post-engraftment showed partial regenerative growth in the implanted area, followed by cell proliferation and angiogenesis.

Advantages and Limitations of Phytogenic Bone Substitute Grafts

Phytogenic materials are natural in origin and abundantly available, but along with them come a few drawbacks. Table 2 gives a gist of the advantages and limitations of various phytogenic materials.

**Table 2:** Advantages and limitations of various phytogenic-based bone graft materials ALP: alkaline phosphatase

Phytogenic materials	Advantages	Limitations
Algipore ^TM^	Good resorbability, large surface area for protein adhesion, and low immunogenicity	Lack of investigations or studies in humans
Gusuibu	Osteoinductive properties and increased alkaline phosphatase activity	A suitable carrier is required to attain complete action, lack of intensive studies in humans and larger animal samples
Icariin	Enhanced osteoblastic activity Inhibits osteoclastic activity	Low bioavailability and short half-life
Naringin	Enhanced ALP activity, increased osteocalcin level, enhanced osteopontin synthesis, and increased osteoblastic cell proliferation	Poor bioavailability, poor water solubility, and the need to be combined with other biomaterials to prevent its degradation
Quercetin	Enhanced osteoblastic cell proliferation, inhibits osteoclastic cell proliferation, promotes antioxidant expression, and inhibits oxidative stress	Low bioavailability and the need to be combined with other materials for sustained release
Kaempferol and Puerarin	Good osteogenic properties	Need to be combined with biomaterials to improve bioavailability
Curcumin	Abundantly available, antioxidant and anti-cancerous properties, and increases the proliferation of osteoblasts	Reduced aqueous solubility, rapid elimination from the system, and inadequate tissue absorption and degradation
Resveratrol, Salvianolic Acids, Ginsenosides, and Ursolic Acid	Naturally available, promotes bone regeneration, and inhibits osteoclasts	Low bioavailability and need a combination of biomaterials
*Morinda citrifolia* fruit extract (noni)	Increased alkaline phosphatase activity and good osteogenic properties	Lack of investigations or studies in humans
Aqueous bark extract of *Salacia chinensis* Apple	Available in abundance and bone regenerative action	Has to be combined with biomaterials for better action and requires further studies

Future of Phytogenic Bone Substitute Grafts

Currently, numerous phytogenic bone grafts and substitutes are available and under investigation. While these materials boast advantages such as abundant availability, optimal bone graft properties, and cost-effectiveness, their application in bone regeneration remains suboptimal. They also present several drawbacks, including low bioavailability, which often necessitates combining them with other biomaterials, subsequently raising costs. Furthermore, certain plant extracts can be toxic to humans when administered in high doses. There's a pressing need to identify a carrier material for these phytogenic components that's reliable, biocompatible, and economical. Only with such advancements can we anticipate the widespread adoption of these bone grafts or substitutes. These challenges underscore the importance of conducting further research on available phytogenic materials. Comprehensive studies, particularly in the field of dentistry, are essential to pinpointing the most effective phytogenic bone graft. As we progress in this domain, it's also crucial to ensure the sustainability and preservation of ecosystems when considering plant sources as bone graft materials.

## Conclusions

Bone grafting remains essential in medical specialties like orthopedics and dentistry. Traditional grafts, such as autografts, allografts, and xenografts, have long been the standard. However, phytogenic materials derived from plants present a promising alternative. Their attributes, including cost-effectiveness, accessibility, and reduced rejection risk, highlight their potential as future bone graft materials. Yet, embracing phytogenic grafts requires thorough research, given challenges like quality control and clinical effectiveness uncertainties. With further exploration, these plant-based materials could significantly impact the future of bone grafting.
